# Access to primary healthcare services among adults with disabilities in Brazil

**DOI:** 10.11606/s1518-8787.2024058005842

**Published:** 2024-10-04

**Authors:** Veronika Reichenberger, Maria Eduarda Lima de Carvalho, Tom Shakespeare, Shaffa Hameed, Tereza Maciel Lyra, Maria do Socorro Velo de Albuquerque, Loveday Penn-Kekana, Christina May Moran de Brito, Luciana Sepúlveda Köptcke, Hannah Kuper

**Affiliations:** I London School of Hygiene & Tropical Medicine International Centre for Evidence in Disability London United Kingdom London School of Hygiene & Tropical Medicine. International Centre for Evidence in Disability. London, United Kingdom; II Universidade Federal de Pernambuco Centro de Ciências Médicas Recife PE Brasil Universidade Federal de Pernambuco. Centro de Ciências Médicas. Recife, PE, Brasil; III Fundação Oswaldo Cruz Instituto Aggeu Magalhães Recife PE Brasil Fundação Oswaldo Cruz. Instituto Aggeu Magalhães. Recife, PE, Brasil; IV London School of Hygiene & Tropical Medicine Department of Epidemiology and Public Health London United Kingdom London School of Hygiene & Tropical Medicine. Department of Epidemiology and Public Health. Maternal and Neonatal Health Group. London, United Kingdom; V Universidade de São Paulo Faculdade de Medicina São Paulo SP Brasil Universidade de São Paulo. Faculdade de Medicina. São Paulo, SP, Brasil; VI Fundação Oswaldo Cruz Brasília DF Brasil Fundação Oswaldo Cruz. Brasília, DF, Brasil

**Keywords:** Health Services Accessibility, Health Services for Persons with Disabilities, Barriers to Access of Health Services, Health Information Systems

## Abstract

**OBJECTIVE:**

To investigate perspectives of people with disabilities in Brazil regarding the access to primary healthcare.

**METHODS:**

In-depth interviews were conducted with 44 individuals with disabilities in Pernambuco, Distrito Federal, and São Paulo between March 2020 and November 2021. These interviews were transcribed, coded, and analysed thematically, using the Levesque framework to identify healthcare access barriers.

**RESULTS:**

Participants expressed a solid understanding of their healthcare needs and existing obstacles. However, individuals with hearing and visual impairments experience challenges because of communication barriers. In Pernambuco, the Community Health Agent was often the initial point of contact for primary care services. Public transportation lacked accessibility, from buses to driver attitudes, posing difficulties for people with disabilities. More accessible transportation and improved urban infrastructure could enhance service access. High medication costs were reported due to limited healthcare unit availability. Communication accessibility issues, inadequate audio-visual resources and equipment were also identified as barriers. Attitudinal barriers among healthcare professionals and subpar home visit services further hinder access.

**CONCLUSION:**

To address these challenges and improve the well-being of individuals with disabilities in Brazil, comprehensive action is essential. This includes leadership, governance, and resource allocation reforms to meet healthcare needs. Initiatives like disability-focused training for service providers, enhanced transportation options, improved information accessibility, and increased support from community healthcare workers can collectively enhance the lives of people with disabilities.

## INTRODUCTION

Globally, 1.3 billion people have disabilities^1^, with at least 17.3 million living in Brazil, comprising 8.4% of the population^2^. Studies have indicated that individuals with disabilities often have worse general health^1,3^, which is partially attributable to existing health conditions and underlying impairments^4^. Furthermore, socioeconomic factors, including age and economic status, contribute to their health disparities^5,6^.

Discrimination and barriers to healthcare access exacerbate these challenges. According to Othero and Dalmaso^7^, in relation to the health of people with disabilities, access was identified as the main need of this population, seen in a broader way, including access to opportunities, movement in the city, and available services.

Brazil’s policy affirms the fundamental right of people with disabilities to high-quality healthcare, emphasizing interdisciplinary teams, suitable infrastructure, communication resources, and assistive devices^8^. The Brazilian Unified Health System (SUS) has played a pivotal role in advancing human rights and diminishing social disparities, notably enhancing access to essential services^9^.

Prior to 2011, healthcare for people with disabilities in Brazil was a notably overlooked aspect of SUS. The turning point occurred when the National Plan for the Rights of People with Disabilities, known as the Living Without Limits Plan, was established through Decree 7.612 in November 2011^10^. This strategic initiative is designed to champion the complete realization of the rights of individuals with disabilities by seamlessly integrating and coordinating policies, programs, and actions. Consequently, the nation embarked on a trajectory of meaningful progress, actively working towards providing essential support for this segment of the population.

Against this historical context, the establishment of the Network of Care for the Health of People with Disabilities was a pivotal step. This initiative was designed with the primary goal of fostering and expanding connections among healthcare services and ensuring access that is characterized by quality, equity, and comprehensive healthcare standards^11^. The innovative structure of the new Network encompasses distinct levels of care, including Primary Care, Specialized Rehabilitation Care, and Hospital and Urgent and Emergency Care. Within each level, services serve as focal points for specific actions in the care of people with disabilities. However, seamless access to these services necessitates active coordination between them, emphasizing the importance of a well-integrated healthcare system.

As outlined in Ordinance GM/MS No. 793/2012, the Primary Care component within the organization of the Care Network designates Basic Health Units as pivotal points for care, encompassing NASF-AB and Dental Care^11^. This approach not only contributes to broadening access but also enhances the quality of care for users with disabilities^11^. Leveraging its extensive reach and proximity to communities, primary care, functioning as care coordinator, assumes a crucial role in championing equitable access for users with disabilities. This ensures tailored care that addresses the specificities and vulnerabilities identified in this demographic.

Presently, a significant challenge persists in the form of inadequate coordination between primary care teams and other components within the Care Network for People with Disabilities, hindering the effective implementation of the ordinance. Examining the guidelines and organizational framework of the Health Care Network (RAS), as recommended in Ordinance 4.279/2010, revealed a pronounced and concerning fragmentation among actions and services within the network^12^. This fragmentation not only signifies a vulnerability but is also evident in the care practices for individuals with disabilities. Noteworthy peculiarities emerge, delving into aspects of the work process consolidated within primary care teams, particularly emphasizing NASF-AB and the Home Care Service (SAD), both crucial support points for the Care Network^13^. Research conducted in São Paulo revealed challenges in healthcare access, which were attributed to deficient infrastructure and healthcare provider stigma^14^. Similarly, children with Congenital Zika Syndrome face significant obstacles, such as stigma and inadequate infrastructure, which hinder their access to vital healthcare services^15^.

Previous research on healthcare access for individuals with disabilities in Brazil has frequently been limited in scope, often focusing on single regions^7,14-19^. Consequently, the objective of this study was to investigate the perspectives of people with disabilities in various regions of Brazil, with a specific focus on the barriers to and facilitators of access to primary healthcare services.

We used the Levesque et al. framework (Figure), which supports the conceptualisation of access to healthcare. The framework consists of five dimensions to access from both the supply and demand sides. Thus, we focused on the patient-centric “demand” aspects of the framework.

**Figure:**
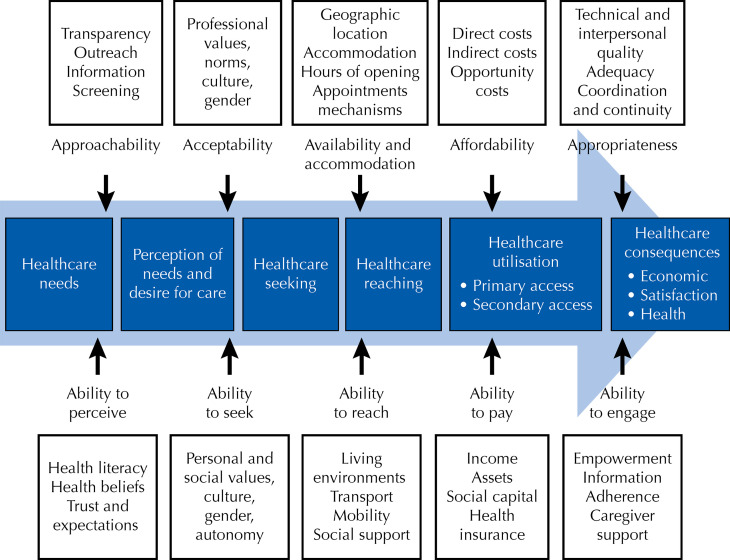
Levesque et al.^20^ framework on healthcare access.

## METHODS

### Overview of Study Design and Setting

In-depth interviews were conducted with adults with disabilities and carers in Arcoverde (Pernambuco state), Brasilia (Distrito Federal state), Santos (São Paulo state) and São Paulo (São Paulo state) (March 2020 to November 2021).

### Sampling and Recruitment

Participants with disabilities were identified through healthcare centres, and snowball sampling was then applied. Some Organisations of Persons with Disabilities (OPDs) were also contacted. Participants were eligible to take part if they were above 18 years old, who reported having ‘a lot of difficulty’ or ‘cannot do’ in one or more of the Washington Group Questions^21^. All participants were approached through telephone or email. We aimed to include a balanced number of participants who self-reported having different disabilities (visual, hearing, intellectual, physical impairment), including men and women, and those older or younger than 65 years.

### Data Collection

#### In-depth Interviews

In-depth interviews were conducted by local researchers, as well as the first author. Although questions about the impact of COVID-19 on healthcare accessibility were posed, they are not aligned with the scope of this paper. The objective of this paper is to comprehend accessibility independent of the pandemic’s influences. All researchers were chosen because of their expertise in the field of public health or psychology and their local knowledge. Two interviewers themselves have a disability.

In-depth interviews were conducted between March 2020 and November 2021, which coincided with the COVID-19 pandemic. Consequently, all interviews in São Paulo, Santos, and Brasilia were conducted remotely, through virtual or telephone calls. The platform was chosen according to the preferences and access availability of each participant. Interviews in Arcoverde were conducted when most restrictions were lifted; therefore, there were a mix of remote and in-person interviews. Participants who were interviewed in person as well as interviewers were required to have no COVID-19 symptoms and to wear a mask during the interview. Most participants were interviewed directly, but interviews with caregivers and family members were used for people with severe difficulties in communicating or understanding. The interview guide was created based on the Levesque framework^20^ and serves as a tool to facilitate the development of interview guides. It includes open-ended questions to aid in exploring additional themes that may emerge.

## Data Analysis

Interviews were transcribed for analysis. Codes were created deductively and inductively within a framework to map the healthcare access experiences of people with disabilities. We used the patient-oriented dimensions of the Levesque framework (Figure). To report this study, we used the Consolidated criteria for Reporting Qualitative research (COREQ) to support us in the different stages^22^. The transcribed interviews were analysed following Bardin^23^ and Kvale’s^24^ hematic cores of meaning approach to unveil deductive categories of access by Levesque et al.^20^. Some inductive categories arose and were kept.

## Ethics

The research was approved by the Ethics Committees of all partner institutions. Participants gave informed consent before taking part in the study. Two consent forms, a more complex form and a simpler form, were created to support understanding. Proxy consent was given for those who found understanding the consent form challenging.

## RESULTS

A total of 44 participants took part in this study, nine in Arcoverde, 16 in Brasilia, six in Santos, and 13 in São Paulo. Most participants interviewed had a physical impairment (24 participants–while 11 had a visual impairment, 4 had a hearing impairment, and 8 had an intellectual impairment). Of these, three had multiple impairments (Table).

**Table:** Overview of participant characteristics.

Region	Total participants	Male	Female	Age ≥ 60	Age < 60	Physical impairment	Visual impairment	Hearing impairment	Intellectual impairment
Arcoverde (PE)	9 (4 interviews given by proxies)	6	3	3	6	1	3	3	2
Brasilia (DF)	16 (5 interviews given by proxies)	7	9	2	14	6 (one with both a physical and an intellectual impairment)	6	1	4 (one with both a physical and an intellectual impairment)
Santos (SP)	6	2	4	2	4	5	1	0	0
São Paulo (SP)	13 (2 interviews given by proxies)	4	9	2	11	12 (2 with both a physical and an intellectual impairment)	1	0	2 with physical and intellectual impairments
Total	44	19	25	9	35	24	11	4	8

This study employed the access dimensions delineated by Levesque et al.^20^, who comprehensively defined access as the opportunity to fulfill health needs, enabling individuals to navigate the steps required to contact and receive healthcare. The framework comprises five dimensions (accessibility, acceptability, availability and accommodation, affordability, and appropriateness), each associated with five corresponding abilities of populations (ability to perceive, seek, reach, pay, and engage). The application of the Levesque framework to comprehend healthcare access for people with disabilities in Brazil yielded inductive categories, which were retained to emphasize a targeted understanding of the barriers faced by this population in the country (Chart).

**Chart:** Overview of the key findings

Themes	Sub-themes
Ability to perceive	Health literacy
	Awareness of personal healthcare needs
Ability to seek	Knowledge of healthcare options
	History of healthcare experience
Ability to reach	Appointments mechanisms
	Logistical support
	Transport
	Physical environment
	Healthcare centre accessibility
Ability to pay	Medication Costs
Ability to engage	Audible and visual accessibility
	Equipment accessibility
	Home visits

### Ability to Perceive (Health Literacy and Awareness of Personal Healthcare Needs)

Many participants described not being aware of any unmet healthcare need. It is important to consider here that, in these cases, participants may not be aware of the real need for healthcare and are therefore starting from a different perspective than those who do. However, others who mentioned specific needs expressed very detailed knowledge about their health, describing detailed accounts of their experiences and treatments. Overall, therefore, the participants appeared to have good knowledge and healthcare literacy and, consequently, a strong ability to perceive their needs. This knowledge came from either previous healthcare visits or family history of healthcare needs.

Some participants had higher healthcare needs and required regular examinations. One participant in Arcoverde (PE) mentioned using a urinary catheter five times a day because he has a neurogenic bladder and intestine.

*“I have a recurring bladder infection, so I need to be cared for closely, or else I can even risk dying.” (Man with physical impairment, 51, PE)*

Participants who had a history of chronic health conditions, such as high blood pressure, depression, and diabetes, also had consistent check-ups and expressed good awareness of the healthcare habits they should adopt. One physically impaired woman in São Paulo reported having check-ups every six months for blood pressure and anemia. Thanks to her check-ups, medical staff were able to identify the onset of thrombosis and treat it before it progressed. Participants frequently reported very good understanding of what led to specific complaints, reporting that doctors were good at explaining their health condition. However, poorer healthcare literacy was mostly reported by hearing impaired participants, visually impaired participants, and intellectually impaired participants, which is reported further under ‘Ability to engage’, as it is linked to communication barriers.

### Ability to Seek (Knowledge of Healthcare Options and History of Healthcare Experience)

Participants reported knowing their primary healthcare options, including where to go and who to seek when this need arises. Some participants, mostly from Pernambuco, reported their “community healthcare agent” (a community health worker) as their first port of call.

The main factor affecting participants’ decision not to seek care, however, was past unsatisfactory experiences with healthcare, including negative attitudes, lack of accessibility, and unmet needs. As expressed by one participant:

*“I prefer to self-medicate because the barriers are so many.” (Physically impaired woman, 39, DF)*

More examples of attitudes and physical accessibility are reported under ‘Ability to reach’ and ‘Ability to engage’.

### Ability to Reach (Appointment Mechanisms, Logistics Support, Transport, Physical Environment, Healthcare Centre Accessibility)

#### Appointment mechanisms

While the waiting time to see a healthcare professional is often long, the consultations tend to be short. Participants reported distress due to long waiting times in facilities and how this affects people differently, according to their impairment type. Wheelchair users struggle with waiting in the same position for long hours, as well as the lack of accessible toilets. A mother of a son with Down Syndrome mentioned the struggle for waiting hours in a hospital waiting area, and her son getting uneasy and stressed, so they occasionally leave before seeing a doctor. A woman who became visually impaired in 2009 explains:

*“It’s important for me that I can be seen by a doctor quickly. I don’t like to wait long periods of time in crowded places, it bothers me. When I had my sight, it was already distressing, but now it’s much worse.” (Woman with visual impairment, 43, PE)*

One visually impaired participant reported the lack of an accessible online booking system leading him not to be able to book appointments on his own. Other visually impaired individuals mentioned booking via telephone or needing someone to book appointments on their behalf.

#### Logistical support

The need for logistical support to reach healthcare services, mostly provided by family members, was reported by all participants with a physical or visual impairment. Family members of participants with intellectual impairment also reported accompanying them to services. This need varied from requiring support in only one specific moment (e.g. putting their wheelchair into a car) or support throughout the entire journey and at the healthcare centre. For participants who are physically impaired, this was reported as one of the first barriers to reach healthcare services.

*“I used to have a heart condition, and I didn’t do the appropriate check-ups because it was hard to find someone to go with me. I need help pushing my wheelchair because I don’t have enough arm strength to do it myself.” (Man with physical impairment, 61, DF)*

Some participants needed support throughout the whole process, from leaving the house to entering a consultation room. As reported by a man with visual impairment in Santos:

*“It’s not just about arriving at my healthcare centre, when I’m there, it’s hard to know what consultation rooms to go to, and there’s no braille in the lifts.” (Man with visual impairment, 48, SP)*

#### Transport

The absence of accessible transportation was predominantly noted by individuals with physical or visual impairments. Among participants with visual impairments in all areas, there was a common sentiment of discomfort when considering independent use of public transportation. Instead, they expressed a preference for utilizing private services, which included options like motorcycle taxis in Pernambuco, or relying on apps such as Uber, as reported in both Distrito Federal and the State of São Paulo.

*“I have my trusted motorcycle taxi driver who takes me where I need to. I have to pay, yes, but at least I know I can trust him, and I feel comfortable. It’s also cheaper than taking cars.” (Woman with visual impairment, 43, PE)*

*“My biggest difficulty is this, going out into the street, […] I always depend on someone, on my husband, my son. And if we need to go anywhere farther away, we always need to pay to go by car using one of those applications.” (Woman with visual and physical impairment, 64, DF)*

There is a lack of appropriately maintained accessible transportation, and broken ramps prevent users from entering public buses. Additionally, bus drivers sometimes do not stop when they see wheelchair users because *“they consider it a hassle to stop and help us on”* reported a physically impaired man in Distrito Federal. This left participants with no or few free transportation options.

*“The electric ramp on buses doesn’t always work. And while they try to get it down, people look at me angrily. That’s why my husband and I walk as far as we possibly can.” (Woman with physical impairment, 41, SP)*

Participants in Arcoverde (PE) did not report on the quality of public transportation because it was scarce. Most people travel by motorcycle taxi or shared vans. The exception was the city of São Paulo, which has a public service called ‘Atende +’ provided by the local government. It is reported to be accessible, easy to book, and very comfortable.

#### Physical environment

The physical environment or urban infrastructure around the healthcare facility was frequently reported as a barrier to healthcare services, mostly by participants with visual or physical impairments.

*“Inside, it’s great, it’s very accessible. But it’s just terrible to get there […] I must walk on the street; I can’t take the sidewalk.” (Man with physical impairment, 38, SP)*

*“In my neighbourhood, unfortunately, I often have to walk on the street. And not to mention that there are sometimes cars parked on the sidewalk, making it totally inaccessible. For you to walk, sometimes you end up having more difficulty on the sidewalks, right, than if you follow the edge of the curb. […] There might be potholes and uneven pavement.”* (Woman with visual impairment, 37, SP)

#### Healthcare centre accessibility

Healthcare centre accessibility was variable. In the state of São Paulo, healthcare centres were reported to have appropriate infrastructure for people with disabilities, having tactile floors, good signage in the clinics, and adequate ramps. Physical accessibility in Distrito Federal and Pernambuco varied. Physically impaired participants in Distrito Federal reported that some health facilities did not have accessible bathrooms, no ramps to enter the centre, or had ramps that were too steep to use on one’s own.

*“I’ve never been inside my local hospital. I can’t even get in! There’s another hospital near us that is also not accessible. There’s a very steep ramp to go up, you can’t even call it a ramp. So, for starters, the health system doesn’t even have accessibility. And nobody cares.” (Woman with physical impairment, 40, DF)*

Participants with visual impairment also mentioned the lack of handrails in healthcare centres, especially when there are stairs, and the difficulty of reaching consultation rooms if they are on their own. A visually impaired woman in Brasilia describes not getting the same quality of care because it is harder for her to reach the appropriate rooms when she is going to a consultation. There is generally no one at the hospital to help her unless she asks, which is why she prefers going with someone.

## Ability to Pay (Medication Costs)

The focus of this study is on SUS services, which are freely available to all Brazilians. The ability of people with disabilities to pay is therefore related to payment for transportation—as reported on ‘Ability to reach’—and out-of-pocket payments for medications.

Some participants reported the non-availability of medication in their healthcare units. Others reported having to pay excessive amounts for medication. There is a very wide range of medications and resources (e.g., orthosis and prosthesis) available through the SUS, but some are not available. A physically impaired participant in Santos (SP) reported:

*“The medication that I have to take daily costs more than what I get paid per month.” (Woman with physical impairment, 41, SP)*

As the above quote indicates, participants experience catastrophic expenditures, which were commonly reported. A participant in Distrito Federal with severe depression and suicidal thoughts reported:

*“I have a physical problem and mental health issues. And the doctor comes to me and says, ‘I can’t give you this medication because you don’t have the money to buy it.’ It’s brutal for me to hear that from someone who deals with health, that they invariably condemn me to be unhealthy because I don’t have money.” (Woman with physical impairment, 47, DF)*

## Ability to Engage (Audible and Visual Accessibility, Equipment Accessibility and Home Visits)

Within the Levesque Framework^20^ ‘Ability to engage’ assesses service quality, ensuring correct treatment and referrals, as well as patients’ ability to decide and engage autonomously. Inductive categories crucial for understanding patients’ ability to engage prominently feature audible and visual accessibility, equipment accessibility, and notably, home visits. Home visits emerged as a pivotal factor that plays a crucial role in either facilitating or hindering the engagement of people with disabilities in the healthcare system in Brazil. This underscores the significance of personalized, at-home healthcare services in ensuring comprehensive and accessible support for this demographic.

### Audible and visual accessibility

Hearing-impaired participants encountered difficulties in effective communication with both healthcare centre staff and professionals. These challenges began in the waiting room, where participants often experienced inadequate notifications regarding their turn for consultations, leading to missed appointments. Furthermore, a significant issue arose from the absence of a shared language because healthcare providers lacked proficiency in Brazilian Sign Language (LIBRAS), and written communication proved ineffective. Consequently, substantial gaps have emerged in the exchange of information and the acquisition of essential healthcare knowledge. Hearing-impaired individuals frequently reported receiving inadequate medication instructions and insufficient information regarding their health requirements.

Visually impaired participants also mentioned gaps in accessibility throughout the healthcare process.

*“Nowadays, if you go to the pharmacy, you find the name of the medicine written in braille in some boxes, but you don’t find the expiration date. I have some medication at home that we leave here, as a precaution, but who knows if they’re expired. […] Another thing that I find difficult about it is when a doctor gives me some tests to do. They tend to give me a referral on a sheet, and when I call to book it, I can’t tell them what’s written on the paper.” (Woman with visual impairment, 37, SP)*

### Another issue reported is that healthcare providers talk to their companions rather than directly to them. As one participant stated

*“I can understand, why don’t they talk to me?” To be honest, I’ve given up. […] They consider us inept.” (Woman with visual impairment, 39, DF)*

She goes on to say that they do not know how to ‘deal’ with her when she goes to consultations on her own and notices the difference in the quality of care from when she goes to an appointment with a family member and how that is worse when she is on her own.

### Equipment accessibility

Some participants had positive experiences with hospital staff members assisting them with inaccessible beds and examination tables. For instance, one woman with physical impairments mentioned receiving help from hospital staff to access a bed for a mammogram. However, many could not undergo the required examinations due to a lack of assistance from healthcare staff, including mammograms, electrocardiograms, x-rays, and smear tests. A physically impaired woman recounted an incident where she requested her doctor’s help for a mammogram, but he declined, citing unauthorized assistance.

### Home visits

A common belief is that home visits serve as a valuable service to assist individuals with disabilities in mitigating the numerous accessibility challenges they face. However, many participants reported that they needed more home visits.

*“Community healthcare agents have never visited anyone’s houses around here. I don’t know if there is a service like that provided by our health centre. I think it would be very important.” (Man with physical impairment, 61, DF)*

As mentioned previously on ‘Ability to seek’, community healthcare agents serve as an important first port of call to support people seeking care, which is mostly reported by people with disabilities in Pernambuco. They tend to be people who know the community, are easy to reach, and are trustworthy.

## DISCUSSION

Our study on healthcare access for Brazilian individuals with disabilities revealed key factors in their healthcare journey. Most participants exhibited strong health awareness, prompting them to seek healthcare support or, for those with past negative experiences, opt for self-medication.

Inadequate physical accessibility at healthcare centres posed another significant issue, marked by the absence of ramps, accessible toilets, and handrails. Visual impairment led to difficulties in appointment scheduling, consultations, and meeting healthcare needs. Confirming our findings, other Brazilian studies have underscored accessibility problems^19,25^. Notably, some participants in one study could not access examinations conducted on inaccessible floors^25^. Research by Girondi et al.^18^ highlighted architectural and layout challenges as primary obstacles to healthcare accessibility, while an analysis of National Census data on Basic Health Units conducted by Santos et al.^26^ revealed nationwide concerns about architectural and communication barriers in primary care. These findings weaken the role of primary care as a coordinator of comprehensive healthcare, compromising access, quality, and effectiveness.

Inadequate accessibility led participants with disabilities to depend on others for healthcare access, underscoring the importance of robust social support networks. This aligns with previous Brazilian studies emphasizing the pivotal role of family members or caregivers in individuals with disabilities’ healthcare journeys. These individuals play vital roles in seeking care, facilitating access, communicating with healthcare professionals, and enhancing treatment outcomes^7,27^.

Outside healthcare facilities, participants faced challenges due to inaccessible urban infrastructure, including irregular sidewalks, potholes, and obstructed walkways. These findings echoed those of other Brazilian studies^14,19,25^. Accessible transportation options were inconsistently available to people with disabilities, prompting their reliance on private services like Uber or taxis. Visually impaired individuals expressed a preference for these services because of safety concerns when walking alone to appointments or the difficulty of using public buses independently. A study in Bahia reported similar findings, highlighting the importance of reliable transportation for maintaining patient follow-up when healthcare centre vehicles were unavailable^28^. Our study emphasized the significance of services like “Atende+” in São Paulo, which alleviate transportation barriers and costs and facilitate the access to healthcare services for individuals with disabilities.

Brazil has strong accessibility laws, including Decree 3,298/99^29^, Law 10,098/2000^30^, and ABNT’s NBR 9050^31, 32^. Nonetheless, our study revealed widespread accessibility challenges that affect individuals with varying disabilities. People with hearing impairments encountered communication barriers because healthcare centre personnel frequently lack basic knowledge of Brazilian sign language (LIBRAS). Moreover, the absence of on-site sign language interpreters led to inadequate information dissemination and follow-up care during the consultations. Prioritizing effective communication is essential for healthcare professionals to provide appropriate and humanized care^17^.

Community healthcare agents play a significant role in facilitating access, although their availability remains limited, which contradicts the objective of the Política Nacional de Atenção Básica^33^ of providing comprehensive healthcare coverage. The insufficient funding largely contributes to the inadequate provision of primary care services in local communities, which is consistent with previous research that identified low home visit rates, especially for individuals with chronic health conditions or physical impairments^34^.

This study identified challenges in providing equitable healthcare for individuals with disabilities in Brazil, underscoring the importance of healthcare provider training in areas such as communication, accessibility, cultural competency, and understanding the specific needs of individuals with disabilities. Addressing these training needs can contribute to a more inclusive and patient-centred healthcare system in Brazil. For example, the participants highlighted both positive and negative experiences regarding equipment accessibility in healthcare settings. Healthcare providers should be trained to assist individuals with disabilities in accessing necessary equipment and ensure that examinations and procedures are feasible for all patients. The findings suggest that individuals with disabilities may feel misunderstood or overlooked by healthcare providers. Training in cultural competency is crucial for healthcare professionals to understand and respect the unique needs, experiences, and perspectives of individuals with disabilities.

### Strengths and Limitations

Strengths and limitations exist in this study, impacting result interpretation. In-depth interviews were conducted by researchers with varying experience levels and training, potentially influencing regional disparities. Additionally, interview methods (face-to-face or online) varied by region, with online interviews posing challenges in rapport building and environmental control. Some Arcoverde interviews were held in the state health department’s local centre, possibly introducing bias. On the positive side, the study included diverse participants in terms of age, gender, and disability type. Researchers with disabilities were involved to enhance rapport and data quality.

## CONCLUSION

This study provides valuable insights into the multifaceted challenges faced by people with disabilities in accessing healthcare in Brazil. It highlights the importance of comprehensive reforms, improved training, and inclusive policies to ensure equitable and accessible healthcare for all individuals.
